# 

**Published:** 1924-10

**Authors:** 


### RECENT APPOINTMENTS.

### Medical Officers, &c.

Coleman, Albert, M.B., Ch.B., House Physician, Royal Infirmary, Manchester; Resident Medical Officer, Ancoats Hospital, Manchester.

Cronk, Herbert Leslie, M.R.C.S., L.R.C.P., Assistant School Medical Officer, Cornwall; Assistant County Medical Officer, Lancashire.

Dormer, P. A., M.D., Assistant Medical Officer, Burton-on-Trent; Medical Officer of Health, Jarrow.

Dorward, W. F., M.B., Ch.B.; Superintendent, St. Luke's Hospital, Haifa.

Franks, Charles, L.S.A.; Deputy and Chief Assistant County Medical Officer, Durham.

Gillies, A. C., M.B., House Surgeon, Royal Infirmary, Edinburgh; Resident Medical Officer, Cumberland Infirmary, Carlisle.

Johnstone, G. G., M.D., Senior Assistant County Medical Officer of Health and School Medical Inspector, Wiltshire; Assistant County Medical Officer, Lancashire.

Ledlie, C., M.B., B.S., F.R.C.S., Assistant M.O., Lambeth Hospital; Senior Resident M.O., Miller General Hospital (Guy's Hospital).

McGarrity, John, M.B., Ch.B., D.P.H., Senior Resident Medical Officer and Bacteriologist, City Hospital, Edinburgh; Resident Medical Superintendent and Assistant Medical Officer of Health, Cardiff.

Muschamp, A. J. I., M.B., Medical Officer of Health, Yeadon, Yorks.

Smith, N. Ross, F.R.C.S.; Resident M.O., Freemason's Hospital and Nursing Home, Chelsea.

Streich, C. I., M.B., B.S., House Physician and Acting-Resident Medical Officer, Miller General Hospital; Resident Medical Officer, Royal Chest Hospital.

Thomas, Melbourne G., M.B., B.S., Assistant Medical Officer, St. Giles Hospital, Camberwell; Superintendent Medical Officer, Pontypridd Union.

Thomson, James, M.B., Ch.B., M.R.C.P., Senior House Physician, Prince of Wales General Hospital; Honorary Assistant Physician for Diseases of Children, Royal Infirmary, Dundee.

Wallis, A. Ransome, M.B., Medical Officer, Kilton Hill Infirmary and Worksop Union; District Medical Officer, Worksop, and Public Vaccinator.

Williams, Howell M., M.R.C.S., L.R.C.P., Tuberculosis Physician, Merionethshire; Tuberculosis Physician, Carmarthenshire.

Wright, Caroline Isobel, M.R.C.S., L.R.C.P.; Assistant Medical Officer, Holland (Lincs.).

### Public Health and Hospital Appointments.

Booth, W. H., Secretary, Jessop Hospital for Women, Sheffield; Secretary, Sheffield Royal Hospital.

Brodie, B. U., Senior Sanitary Inspector, Brigg; Senior Sanitary Inspector, Boston.

Bromley, J. W., Assistant Sanitary Inspector, Doncaster; Assistant Sanitary Inspector, Margate.

Crawford, Louisa G. R.; Health Visitor, Darlington.

Dawkins, Susan L.; Health Visitor and School Nurse, Exeter.

Delahunty, Honora, C.M.B.; Matron, Maternity Home, Bromborough Pool, Birkenhead.

Franklin, Kathleen, Matron and Housekeeper, Fitzherbert Terrace School, Wellington, New Zealand; Matron, Convalescent Home, Kenilworth.

Gaddy, Catherine; Superintendent, Queen Victoria's Jubilee Institute, Scunthorpe.

Goulden, L. M.; Health Visitor and School Nurse, Leigh.

Hall, Ruth, A.R.R.C.; Matron, District Hospital, Wellington.

Hipkiss, Lily; School Nurse, Wolverhampton.

Jenkins, K., A.R.R.C.; Matron, Bartlet Convalescent Home, Felixstowe.

Lindsay, Elizabeth O., Matron, Clark Hospital, Largs; Matron, Northern District Infectious Diseases Hospital, Ayrshire.

Maguire, A. V.; Matron, Bangor Hospital, co. Down.

Martin, G. C.; Sanitary Inspector, Warmley.

Mellor, J. W., Assistant Sanitary Inspector, Barnsley; Chief Sanitary Inspector, Dewsbury.

Morris, Mary H., Matron, Friarton Infectious Diseases Hospital, Perth.

Noble, T.; Surveyor and Sanitary Inspector, Sowerby Bridge.

Nurse, Ruth, District Nurse-Midwife, High Wycombe; Health Visitor and School Nurse, Berkshire.

Reynolds, Alexander; Sanitary Inspector, Doncaster.

Smith, Fanny, Assistant Matron, Royal Orthopædic and Spinal Hospital, Birmingham; Matron, Woodlands Orthopædic Hospital, Birmingham.

Stewart, Mary J., Matron, Preston, Fulwood and Longridge Joint Hospital; Matron, Smallpox Hospital, Sutton, Hull.

Wakefield, Charlotte; Matron, Convalescent Home for Children, Weston-super-Mare.

Wheater, Agnes A.; Matron, Maternity and Babies' Hospital, Salford.

Wilkinson, Lilian; Matron, Lambert Memorial Hospital, Thirsk.

### ANSWERS TO CORRESPONDENTS.

Incurables.—The Hostel of God, 29 North Side, Clapham Common, S.W. 4; St. Joseph's Hospital for Incurables, Burlington Lane, Chiswick, W. 4. Both of these homes are under the care of the Sisters of St. Margaret's, East Grinstead.

Secretary.—The Conference was held under the auspices of the Labour Party. A report appeared in our June issue.

P. S. E.—The address of the Chartered Society of Massage and Medical Gymnastics is 157 Great Portland Street, W. 1.

Provincial.—The People's League of Health was founded and is organised by Miss Olga Nethersole. Its object is "The raising of the standard of health of the British Empire." Its address is 12, Stratford Place, W. 1.

### Managerial Notices.

*All letters on Business matters must be addressed to the Manager, "The Hospital and Health Review," 28 and 29 Southampton Street, London, W.C. 2.*

RATES FOR SUBSCRIPTION (payable in advance).

| | | |
|---|---|---|
| Three Months (including Postage) | .. .. | 1s. 9d. |
| Six Months do. | .. .. | 3s. 6d. |
| Twelve Months do. | .. .. | 7s. 0d. |

**It is especially requested that remittances be made by Cheque or Postal Order and not Stamps.**

Subscriptions, which may commence at any time, are payable in advance. Cheques and Post-Office Orders should be crossed Westminster Bank, Covent Garden Branch, and made payable to the Manager of THE HOSPITAL AND HEALTH REVIEW.

***Advertisements.*** Scale of charges and particulars of special positions may be obtained on application to the Manager.

*SUBSCRIPTION FORM.*

*Please send me* THE HOSPITAL AND HEALTH REVIEW *for*............ *months, commencing with your issue of* ..............., *for which please find enclosed* ........

*Name* ..............................

*Address* ..............................

..............................

*Date* ..............................

*To*.... ..............................*Newsagent*

